# Slovakia and its environmental transformation: measuring environmental attitudes using the new ecological paradigm

**DOI:** 10.3389/fpsyg.2024.1320451

**Published:** 2024-04-03

**Authors:** Lenka Marcineková, Martina Štěrbová, Jozef Výbošťok, Iveta Hajdúchová, Blanka Giertliová, Rastislav Šulek, Zuzana Sarvašová, Jaroslav Šálka

**Affiliations:** ^1^Department of Forest Economics and Policy, Faculty of Forestry, Technical University in Zvolen, Zvolen, Slovakia; ^2^Department of Forest Management, National Forest Centre – Forest Research Institute, Zvolen, Slovakia; ^3^Department of Forest Harvesting, Logistics and Ameliorations, Faculty of Forestry, Technical University in Zvolen, Zvolen, Slovakia

**Keywords:** attitude, environment, environmental transformation, new ecological paradigm, public perception

## Abstract

Many scientists have studied the relationship between society and the environment. The New Ecological Paradigm (NEP) has been repeatedly used for the analysis of public environmental attitudes, as the public pays increased attention to the environmental issues, sustainability, or the climate crisis. Our paper deals with the use of the NEP to study and identify public environmental attitudes in the Slovak Republic. We discovered a deviation of our results from the NEP, as we identified altered environmental attitudes, which we find acceptable, as environmental attitudes are a delicate set of values encompassing different, yet interrelated facets. According to our results, we were able to classify our respondents into those with technocentric, ecocentric, and resiliocentric attitudes, while up to 70% of respondents hold the ecocentric attitude. These findings could be influenced by the fact that no significant changes in the population’s lifestyle have been required up to now. The NEP has proven to be an important predictive tool in assessing public environmental attitudes to determine readiness for environmental transformation. Nevertheless, we recommend using a combination of analysis that includes evaluating the impact of demographic factors to achieve higher-quality results.

## 1 Introduction

The environment has always played an essential role in human history. That is why many social scientists have investigated the relationship between people and the environment and the public perception of environmental problems (Dunlap et al., 2000; Hosseinnezhad, 2017). Citizens, as members of society, have great potential to influence policymaking, as their attitudes towards the environment are influencing the management of the natural environments of our societies. While conceding that these attitudes have similar features, they have also been established to be often differing and contradictory among the public (Grendstad and Selle, 2000; Aggestam, 2015). We propose that it is important to study these public attitudes, as the decision-making process of citizens is based on them (Schultz and Zelezny, 1998; Schwartz and Boehnke, 2004).

The New Environmental Paradigm (which is now called the New Ecological Paradigm—the NEP) was developed for this purpose in 1978, and it enabled us to understand people’s attitudes toward the environment and their perception of environmental problems. However, the environmental problems that humankind was facing at that time had evolved over the years. Nowadays, environmental problems have become less local, therefore endangering life on a global scale, less noticeable, whereas the origin of these problems has become more ambiguous (Stern et al., 1992; Dunlap et al., 2000). With this in mind, researchers focusing on public attitudes towards environmental problems have started to pay increasing attention to these “attitude objects” as Dunlap et al. (2000) describe them.

The fact, that people started to recognize the effects of human activities on the environment, and that sustainability and sustainable development became acknowledged necessities, indicates that our beliefs and worldviews are subject to re-evaluation (Milbrath, 1984; Dunlap et al., 2000). Therefore, it is not surprising to see more ecologically oriented worldviews emerging along with efforts to measure “ecological consciousness” (Ellis and Thompson, 1997), “anthropocentrism” (Chandler and Dreger, 1993), and “anthropocentrism versus ecocentrism” (Thompson and Barton, 1994).

According to evolving environmental problems, the NEP scale has also had to be altered. Based on the original NEP scale, which consisted of three facets (fragility of natural balance, existence of the limits to growth, humans’ right to rule over nature), Dunlap et al. (2000) have developed a revised NEP scale, which we will use in this paper. The revised NEP scale takes into account the changing nature of environmental problems, increasing awareness of these problems, removes outdated sexist terminology (mankind = humans), and at the same time is able to capture beliefs concerning the relationship between humans and the environment (Ntanos et al., 2019). Compared to the original NEP scale, the revised one detects five environmental beliefs: namely the reality of limits to growth, anti-anthropocentrism, the fragility of nature’s balance, the rejection of excemptionalism, and the possibility of an eco-crisis (Dunlap et al., 2000). Furthermore, to identify the environmental belief, 15 questions are used, eight of which are pro-NEP oriented and seven of which are anti-NEP oriented (Ntanos et al., 2019). The formulation of these questions ensures that a pro-ecological worldview is indicated by agreement with eight of the questions and by disagreement with the remaining seven questions (Dunlap et al., 2000). Over the years, the NEP has developed into a widely used, popular measure of environmental concern, and is seen as indicating a pro-environmental orientation (Dunlap et al., 2000).

The revised NEP scale is widely used by many researchers whether they are focusing on a country analysis (e.g., Edgell and Nowell, 1989; Widegren, 1998; Bostrom et al., 2006; Hosseinnezhad, 2017; Ntanos et al., 2019), an ethnic analysis (e.g., Caron, 1989), or a communities’ farmers (e.g., Albrecht et al., 1982; Schultz and Zelezny, 1998; Bechtel et al., 1999; Kopnina, 2011).

There is a growing focus on environmental awareness and attitudes in the region. The youth in Poland see themselves as being environmentally conscious. However, there is a prevailing sentiment that their engagement with environmental issues is at an average level and they are not eager to change their lifestyles or actively improve the quality of their environment (Lisowski et al., 2022).

Furthermore, there is an increasing emphasis on environmental education in the region, with a particular focus on children in countries such as Hungary and the Czech Republic. Building on Strong’s (1998) notion that environmental awareness develops in childhood, there has been a noted positive impact of environmental education on young citizens in the Czech Republic, as outlined by Cincera et al. (2022). This research also found that when younger generations feel empowered to participate in decision-making processes, they are more likely to make environmentally conscious choices and take actions. In Hungary, it was observed that children’s inclination towards environmental protection is often rooted in their affection for animals, as suggested by Alfoldi and Alfoldi (2019). In Slovakia, environmental education for young students is not clearly defined and largely depends on individual teachers. Currently, only a limited number of elementary schools in Slovakia are offering such education, indicating a need for the development of a structured system for environmental education in the country, as highlighted by Piscová et al. (2023).

Viewing the region from a pan-European standpoint, it appears to be progressing slowly towards environmental awareness, as noted by Urban and Kaiser (2022). Poland, along with Slovakia, Hungary, and the Czech Republic, ranks at the lower end in terms of public pro-environmental orientation, placing them among the bottom eight countries. This is also reflected in the low inclination towards green consumption within these nations.

Slovakia has implemented various strategies to address environmental issues, particularly climate change and its impact on the environment. The country is focused on becoming more sustainable, as evidenced by the national strategy entitled “*Greener Slovakia – The Strategy of the Environmental Policy of the Slovak Republic until 2030.”* This strategy recognizes the adverse effects of climate change and offers recommendations for combating it. Additionally, Slovakia has published the *Integrated National Energy and Climate Plan for 2021–2030*, which aims to reduce greenhouse gas emissions in the energy sector as part of efforts to combat climate change. The country has also developed the *Low-Carbon Development Strategy of the Slovak Republic until 2030 with a View to 2050*, which takes a comprehensive cross-sectoral approach to addressing climate change. Moreover, Slovakia is aligning itself with European trends in bioeconomy development through the *Strategy for bioeconomy in Slovakia – The contribution of Slovak bioeconomy for the strategic plan of the common agricultural policy 2021–2027*. This demonstrates the country’s commitment to sustainable and environmentally friendly practices in agriculture and beyond.

Given the above, we find the revised NEP scale to be a suitable tool to measure the environmental worldview of the Slovak public, as it is a globally accepted method allowing us to compare our results with the results of other researchers. Environmental awareness is increasing throughout the general public in Slovakia, and people are opting for the use of materials with a less negative impact on the environment (Paluš et al., 2012; Kaputa et al., 2018; Navrátilová et al., 2020; Musova et al., 2021), yet the NEP has not been used to identify public environmental attitudes in Slovakia. Nevertheless, whether people act based on this preference depends mostly on their attitude towards the environment and environmental problems, as their behavior is affected by their attitudes. For this reason, we find it necessary to investigate the environmental attitudes (worldviews) of people in the Slovakian public.

Therefore, our main objective is to identify Slovak citizens’ environmental attitudes using the NEP approach, as a supporting predictive method useful for identifying public perceptions of the environment and environmental problems. Understanding public attitudes towards the environment will render the decision-making process more effective, as well as the communication of these decisions to the public. We find it important to look at citizens´ preferences regarding the environment, together with acknowledging the place their behavior is coming from—their attitudes.

## 2 Materials and methods

### 2.1 Data collection

Data collection was performed in Slovakia in September 2020. It was conducted as a part of the POLYFORES project, which examined the perception of the importance of forests. The survey was conducted by the private company Go4insight, which specializes in public opinion polling. Data collection was carried out via the Internet from a panel created by the agency. The questionnaire was presented in the Slovak language. The Slovak questionnaire was produced by means of translating the original English version. To achieve this, we utilized semantic translation in conjunction with adaptation, in order to ensure that the text captures the syntactic and semantic structures of the original document. In certain instances, alterations were required to identify appropriate equivalents in the Slovak language. By doing so, we were able to effectively convey the same message as the original text, whilst maintaining the context and taking into consideration the cultural norms of Slovakia. The questionnaire consisted of six sections with a different focus—forests and their importance, benefits provided by forests, forest management priorities, people’s relationship with the environment, basic values, and general information about respondents. In total, we collected questionnaires from 1,000 respondents. For the data collection, an online panel was used organized by the Go4Insight company. All our models were estimated using panel data methods in order to control the heterogeneity and the collinearity among the variables (Baltagi, 2005). In total, 33 questionnaires were excluded from the dataset because of incorrect completion; therefore, the sample consists of 967 respondents. The sample structure is displayed in Table 1.

**Table 1:** Demographic characteristics of sample.

	Frequency	Percentage
*Sex*		
Male	456	47
Female	511	53
*Age*		
18–30	211	22
31–40	233	24
41–50	237	25
51–65	286	29
*Education*		
Elementary	19	2
High school without graduation	174	18
High school with graduation	484	50
University	290	30

In this paper, we focused on the questions from the fourth section which deal with people’s relationship with the environment based on the NEP scale. The NEP scale items were designed to tap into five hypothesized facets of an ecological worldview. These are displayed in Table 2, and include the reality of limits to growth (Q1, Q6, Q11), anti-anthropocentrism (Q2, Q7, Q12), the fragility of natural balance (Q3, Q8, Q13), the rejection of human exceptionalism (Q4, Q9, Q14), and the possibility of an eco-crisis (Q5, Q10, Q15) (Dunlap et al., 2000). We measured the NEP scale variables using a five-point Likert scale, and the scale items were coded from 1 = “strongly disagree” to 5 = “strongly agree,” where 3 is considered “neutral.” The negative phrases were subsequently reverse-coded.

**Table 2:** NEP scale items.

Facets of the ecological worldview		Statement
Limits to growth	Q1	We are approaching the limit of the number of people the earth can support.
	Q6	The earth has plenty of natural resources if we just learn how to develop them.
	Q11	The earth is like a spaceship with very limited room and resources.
Anti-anthropocentrism	Q2	Humans have the right to modify the natural environment to suit their needs.
	Q7	Plants and animals have as much right as humans to exist.
	Q12	Humans were meant to rule over the rest of nature.
Fragility of nature’s balance	Q3	When humans interfere with nature it often produces disastrous consequences.
	Q8	The balance of nature is strong enough to cope with the impacts of modern industrial nations.
	Q13	The balance of nature is very delicate and easily upset.
Rejection of human exceptionalism	Q4	Human ingenuity will ensure that we do not make the earth unliveable.
	Q9	Despite our special abilities, humans are still subject to the laws of nature.
	Q14	Humans will eventually learn enough about how nature works to be able to control it.
Possibility of an eco-crisis	Q5	Humans are severely abusing the environment.
	Q10	The so-called “ecological crisis” facing humankind has been greatly exaggerated.
	Q15	If things continue on their present course, we will soon experience a major ecological catastrophe.

### 2.2 Data analysis

In order to achieve the objectives of this research, we used a descriptive statistics tool, one-way analysis of variance, Cronbach’s alpha, exploratory factor analysis, and Pearson correlation. Descriptive statistics were employed to understand basic characteristics. We used a one-way analysis of variance to investigate the differences between sex, age category, and education to obtain a total NEP score average. In order to obtain the major differences, we used the Tukey HSD *post hoc* test. Cronbach’s alpha tests were used to verify internal consistency in the current research. Principal component analysis was used to investigate the extent to which the pattern of responses among the samples were consistent with the hypothesized structure of the NEP. The relationship of the component to age, sex, and education was determined by Pearson correlation.

## 3 Results

The results presented in Table 3 show no significant difference between male (*A* = 3.49) and female (*A* = 3.50) respondents in terms of their total NEP scores. On the other hand, one-way ANOVA reveals a significant difference between age categories. The Tukey HSD *post hoc* test shows that major differences were found between the 18–30 age category (*A* = 3.44) and the 51–65 age category (*A* = 3.52). One-way ANOVA further shows that the most significant differences were between types of respondents’ education in terms of their total NEP scores. The Tukey HSD *post hoc* test shows the major differences between respondents with high school education without graduation (*A* = 3.57) and respondents with high school education with graduation (*A* = 3.49), and also between respondents with high school education without graduation (*A* = 3.57) and respondents with university education (*A* = 3.46).

**Table 3:** The results of one-way analyses of variance with total NEP score average.

Category	Sub-category	Total NEP score average	*F*	sig.
Sex	Male	3.49	0.1	0.7476
	Female	3.50		
Age category	18–30	3.44	2.77	0.0399
	31–40	3.48		
	41–50	3.51		
	51–65	3.52		
Education	Elementary	3.50	4.059	0.0068
	High school without graduation	3.57		
	High school with graduation	3.49		
	University	3.46		

Using principal component analysis (PCA) we investigated the extent to which the pattern of responses among the samples was consistent with the hypothesized structure of the NEP, in the same way that other authors did who used similar data (Ogunbode, 2013; Ntanos et al., 2019).

First, we verified the internal consistency of the current research using Cronbach’s alpha tests. A Cronbach’s alpha value equal to 0.562 was obtained, which showed an acceptable level of reliability at the lower limit of the range because an alpha of lower than 0.5 represents unacceptable internal consistency. We then calculated Kaizer–Meyer–Olkin’s measure of sampling adequacy (KMO = 0.871) and Bartlett’s test of sphericity (*p* = 0.000). According to the results, data were acceptable for PCA. Other results which we required were anti-image matrices that showed measures of sampling adequacy (MSA). In all cases, MSA was greater than 0.5 which means that all cases are appropriate for PCA. After verifying the suitability of the data, we approached the extraction of factors. Because the results for no rotation solution in implementation of PCA were difficult to interpret, and variables belonged to several factors simultaneously, we opted for varimax rotation which showed better interpretation. Based on the number of components with eigenvalues higher than 1 (which in combination explained 49% of the variance in the data), we were able to identify three components in line with the NEP (Table 4). The cluster of NEP items on the first component, explained by 27.8% of the variance, indicates that this component captures all of the NEP facets.

**Table 4:** Total variance explained by components.

Component	Initial eigenvalues		
	Total	% of Variance	Cumulative %
1	4.166	27.772	27.772
2	2.099	13.991	41.763
3	1.142	7.614	49.377
4	0.865	5.766	55.143
5	0.790	5.268	60.411
6	0.743	4.955	65.366
7	0.682	4.544	69.910
8	0.678	4.519	74.429
9	0.648	4.322	78.751
10	0.611	4.076	82.827
11	0.584	3.894	86.721
12	0.554	3.694	90.415
13	0.503	3.350	93.765
14	0.473	3.152	96.918
15	0.462	3.082	100.000

The results explaining how the NEP scale items are grouped into three extracted components based on their loadings are presented in Table 5.

**Table 5:** Principal component analysis of NEP items with varimax rotation.

NEP Scale items	Components		
	1	2	3
Q1: “We are approaching the limit of the number of people the earth can support.”		0.745	
Q2: “Humans have the right to modify the natural environment to suit their needs.”	0.663		
Q3: “When humans interfere with nature it often produces disastrous consequences.”			0.567
Q4: “Human ingenuity will ensure that we do not make the earth unliveable.”	0.666		
Q5: “Humans are severely abusing the environment.”			0.602
Q6: “The Earth has plenty of natural resources if we just learn how to develop them.”			0.674
Q7: “Plants and animals have as much right as humans to exist.”			0.678
Q8: “The balance of nature is strong enough to cope with the impacts of modern industrial nations.”	0.693	−0.309	
Q9: “Despite our special abilities, humans are still subject to the laws of nature.”			0.565
Q10: “The so-called ‘ecological crisis’ facing humankind has been greatly exaggerated.”	0.610		
Q11: “The Earth is like a spaceship with very limited room and resources.”		0.704	
Q12: “Humans were meant to rule over the rest of nature.”	0.653		−0.326
Q13: “The balance of nature is very delicate and easily upset.”			0.507
Q14: “Humans will eventually learn enough about how nature works to be able to control it.”	0.664		
Q15: “If things continue on their present course, we will soon experience a major ecological catastrophe.”		0.589	

Table 6 shows the results of the agreement and disagreement statements’ cumulative percentage, as well as the neutral statements’ percentage with mean, median, and standard deviation. The results showed that the strongest anthropological orientation is displayed in the Component 1. The average response was 2.39. On the other hand, the most biocentric orientation is displayed in the Component 3. For these reasons, we named these components technocentrism (Component 1), resiliocentrism (Compotent 2), and ecocentrism (Component 3). The average response for ecocentrism was 4.34. The average response for resiliocentrism (3.99) was within the range of technocentrism and ecocentrism. The overall average has a value of 3.49.

**Table 6:** NEP items with frequency, mean, median, and standard deviation of response.

NEP scales dimensions	NEP scales items	Cumulative percentage			Mean	Median	S.D.	Percentage
		A	*N*	D				
Technocentrism	Q2	59.05	23.68	17.27	2.33	2.00	1.12	4%
	Q4	37.85	40.12	22.03	2.73	3.00	1.12	
	Q8	55.63	25.65	18.72	2.40	2.00	1.20	
	Q10	55.84	23.27	20.89	2.41	2.00	1.21	
	Q12	70.32	18.51	11.17	1.95	2.00	1.11	
	Q14	48.09	30.09	21.82	2.55	3.00	1.19	
Resiliocentrism	Q1	63.50	23.27	13.23	3.77	4.00	1.09	26%
	Q11	73.01	15.82	11.17	3.94	4.00	1.06	
	Q15	83.04	11.07	5.89	4.25	4.00	0.94	
Ecocentrism	Q3	87.28	6.72	6.00	4.43	5.00	0.95	70%
	Q5	89.97	4.96	5.07	4.50	5.00	0.90	
	Q6	80.35	12.00	7.65	4.18	4.00	0.98	
	Q7	86.66	7.34	6.00	4.40	5.00	0.93	
	Q9	81.70	13.24	5.06	4.25	5.00	0.92	
	Q13	84.90	10.13	4.97	4.31	5.00	0.87	
Mean total NEP score		56.15	17.73	26.12	3.49	4.00	1.04	

The newly emerged components can represent three attitudes towards the environmentalist on the items they consist of (see Table 6). Technocentrism is a typical anthropocentric attitude towards the environment, as the environment is perceived as a producer of resources necessary to meet the needs of society. Humans have much power to manage but also to protect the environment using technology and innovation, as the prevailing opinion is that all environmental issues can be solved using scientific knowledge, and technology. This attitude is characteristic of 4% of our respondents. An identified opposing attitude, with 26% representation among the respondents, is resiliocentrism, which is a part of biocentrism. Resiliocentric people perceive the environment to be a closed system that is able to absorb a certain level of external impacts without disruption. In this case, attention is paid to the natural limits of the environment, and the scarcity of natural resources is acknowledged. The last identified attitude, ecocentrism, is a part of a biocentric attitude toward the environment, as well, with humans perceived as being an equal part of it. For ecocentric people, who constitute 70% of our respondents, empathy and respect towards all parts of the environment are characteristic, as the environment is perceived as more than just a producer of important resources.

The relationship of the component to age, sex, and education is determined by Pearson correlation, according to which no correlation has been found among the respective components.

## 4 Discussion

Many researchers have used the revised NEP scale for their analyses, whether focusing on a country (e.g., Edgell and Nowell, 1989; Widegren, 1998; Bostrom et al., 2006; Hosseinnezhad, 2017; Ntanos et al., 2019), ethnicities (e.g., Caron, 1989), or even farmers (e.g., Albrecht et al., 1982), school children (Kopnina, 2011) and university students (e.g., Schultz and Zelezny, 1998; Bechtel et al., 1999). In these studies, as Dunlap et al. (2000) stated, a relatively strong endorsement of NEP beliefs across various samples was predominantly discovered. As expected, people belonging to environmental organizations scored higher on the NEP scale compared to those who were not members of such organizations (Edgell and Nowell, 1989; Pierce et al., 1992).

In contrast to the five environmental attitudes defined in the NEP (Dunlap et al., 2000), our results show three slightly altered attitudes. However, these attitudes are still in line with NEP, as they also consist of anthropocentric and biocentric approaches (López-Bonilla and López-Bonilla, 2016). As a representative of an anthropocentric approach and the least represented attitude among the respondents (4%), technocentrism revolves around humans and their competence, knowledge, and skills. Within this environmental attitude there is a common belief that humans have the ability to solve all environmental problems using science, technology, and innovation. At the other end of the spectrum, 70% of our respondents constitute a representative of biocentrism with an ecocentric environmental attitude. In this case, humans are not perceived as a core of the attitude, but as an equal part of the ecosystem respecting every part of the environment. The last identified environmental attitude is resiliocentrism represented by 26% of respondents, which is partly related to ecocentrism, as it also lies within the biocentric attitude. Resiliocentrism is characterized by respect of the environment as a closed system able to eliminate a certain proportion of humans’ impact, while acknowledging the natural biological limits of the ecosystem. Our results confirm a study in which Van den Born et al. (2001) argue that the public in Europe (but it also implies for the United States of America) has developed a strong environmental inclination that often crosses into “biophilia.”

Comparing these attitudes with the NEP methodology, we can see that technocentrism mostly combines aspects of anti-anthropocentrism and a rejection of human exceptionalism, while the answers suggest the opposites—this means that people are leaning toward the anthropocentric orientation. The ecocentric attitude is composed of a mix of each NEP facet with a strong biocentric orientation. The strongest overlap between NEP methodology and our identified attitudes can be found in resiliocentrism and the limits of growth.

However, the NEP is not always a recommended tool to measure public environmental attitudes. The NEP has proven to be slightly unstable when used in several contexts, as the socio-demographic factors of respondents seem to have an impact on it (Brennan et al., 2014), which is partially in line with our analyses, as we found out that age and level of education are significantly affecting the environmental attitudes of our respondents. Also, the NEP appears to focus heavily on aspects of anthropology and social vision, while neglecting other important dimensions. Additionally, NEP shows minimal consideration towards the spirituality of the participants (Lockwood, 1999; Van den Born et al., 2001). Some authors argue that the separation of spirituality from the environment could lead to a materialistic view of nature, even in cases where humans are perceived as an integral part of the natural world (White, 1967; Zweers, 2000; Taylor, 2010). It also demonstrates a good item-fit statistics. However, there is a shared view that there is room for improvement, especially in terms of eliminating redundant items and incorporating new items that effectively capture the entire spectrum of environmental attitude. There is also an argument that the NEP exhibits bias against conservative environmentalism and falls short in its ability to predict environmentally protective behavior. Other research, on the other hand, discovers that the NEP does not inherently contain items of low quality or constitute a poor overall scale (Sparks et al., 2022). It also provides a standardized way to assess individuals’ environmental attitudes, enabling researchers to gain insights into people’s views on the environment. It also allows for comparative analyses across different demographics, locations, and time periods, helping to identify trends, patterns, and shifts in environmental attitudes. Moreover, the predictive power of the NEP is widely recognized, making it a valuable tool for understanding and promoting sustainable behaviors and actions (Stern et al., 1995; Ntanos et al., 2019; Derdowski et al., 2020).

Unsurprisingly, views on the effect of socio-demographic factors on public environmental attitudes differ among authors. According to one group (Uysal et al., 1994; Lück, 2000; Luo and Deng 2008), the influence of socio-demographic factors on environmental attitudes is insignificant. Another group have identified a significant correlation between socio-demographic factors of respondents (such as age and education) and their environmental attitudes (Van Liere and Dunlap, 1980). The effect of respondents´ demographics on the NEP is also confirmed by Luo and Deng (2008). The study by Franzen and Meyer (2008) confirms the effects of income on the level of environmental concern. Individuals with higher income show a higher level of environmental concern; this is also applicable in the context of countries. Thus, wealthier countries, in general, show a higher level of concern about the environment. The wealth of an individual often represents the level of education, which has also already been established to affect environmental attitude, according to a study by Maleki and Karimzadeh (2011) and as confirmed by our results.

On the other hand, gender has not been proved to affect the NEP according to our analyses. This is contrary to a study by Hosseinnezhad (2017), who used the NEP to analyze the attitude of citizens of Iran towards the environment. In this study, significant differences were found between men and women’s attitudes to environmental problems. Women tend to incline toward anti-anthropocentrism more than men; they believe that people are not meant to rule over nature or modify the environment to meet their needs.

Grúňová et al. (2018) have found specific variations that arise from cultural features, religious viewpoints, attitudes towards humans´ place in nature, and low awareness of the impacts that human activities have on natural resources. Further, according to some authors, the NEP omits the spirituality of citizens, thus separating concepts of spirituality from the environment. This leads to a rather materialistic perception of the environment, even in the case of ecocentric attitudes (White, 1967; Zweers, 2000; Taylor, 2010).

The fact that the 8ublicc adopts different attitudes to the environment must be considered as an environmental transformation process. Ignoring this fact could lead to the failure of any communication strategy. However, it is also necessary to optimize the communication and promotion of this issue, so that it reflects various environmental attitudes of the public, in which case, a mutual understanding can be achieved (Bekessy et al., 2018; Jax et al., 2018; Ainscough et al., 2019).

Based on what we know in this particular case – namely that the majority of the Slovak public adopts an ecocentric environmental attitude – it is necessary to optimize a communication strategy, but also specific environmental issues, accordingly. Based on our results, one can assert that the public adopts a positive attitude towards the environment, while it cares about its protection and enhancement; this indicates that the public cares more about the well-being of society than its own. If decision-makers, scientists, or the representatives of practice are interested in communicating with the public more effectively, the knowledge of public preferences is not sufficient, they also need data and information about the public’s environmental attitudes. This approach has the potential to reduce uncertainty about public preferences, as well as increasing the acceptance of proposed solutions to various environmental problems. Nevertheless, the use of public knowledge of environmental attitudes should work as a complementary method, and not as the only source of information. The benefits of this approach are clear when demographic indicators reach their natural limits in explaining the links between citizens’ values and preferences. Yet, the effect of socio-demographic factors on the NEP is confirmed by several studies (Uysal et al., 1994; Lück, 2000; Luo and Deng 2008; Brennan et al., 2014). For this reason, we see a combination of the NEP with an analysis of socio-demographic factors (e.g., gender, age, income, family status, urban/rural residence, religion, political orientation, personal values etc.), as the optimal methodological approach. We believe that a combination of both methodologies can achieve results of a higher quality.

Even though the majority of our respondents lean toward ecocentrism in theory, the actual transformation of people’s behavior remains questionable. This is also confirmed by a long-term increase in transportation emissions over the last years in Slovakia (Slovak Hydrometeorological Institute, 2021). This is also proved by Musova et al. (2021), who discovered that consumers in Slovakia are very willing to purchase environmentally friendly products, yet they do not consider their transport-related behavior to be as important as their consumption. Therefore, it is also reasonable to suggest that the high level of pro-environmental attitudes might be affected by the fact that it has not yet been necessary for the public to significantly change its behavior in line with sustainable principles. Nevertheless, it is a common belief that having a pro-environmental attitude together with implementing environmental activities increases the prospects of pro-environmental behavior (Musova et al., 2021).

Rather than stating that the NEP is not applicable, we find that the differences occur due to the different sample characteristics, as the data was collected using an online panel. We decided to use panel data, as panels have been widely used as a tool in analyzing public opinion about various issues for decades, and to ensure the required sample size and structure. Using panel data certainly has many advantages, as it allows us to design complicated behavioral models and to analyze the effects of individual actions or policies, as well as enabling us to apprehend the essential forces affecting the outcome (Hsiao, 2003). Also, the social and environmental landscape has undergone significant transformations since the inception of the NEP. One of the most notable changes has been the increasing prominence of climate change as a critical and urgent issue. The growing awareness of the impacts of climate change has catalyzed shifts in public attitudes and behaviors toward environmental challenges. Individuals and societies are now more conscious of the need to reassess their consumption patterns, lifestyles, and overall approach to sustainability. This heightened awareness and emphasis on climate change mitigation and adaptation could potentially influence the outcomes and interpretations of studies like ours, leading to variations in results compared to the original methodology. Nevertheless, issues have also been identified concerning the use of this type of data. The main problem is the fact that when people are being frequently interviewed about similar topics it is bound to affect their opinion and perceptions of these topics (Hsiao, 2003). For this reason, we find that using an online panel is one of the limitations of the research. Another limitation can be found in the national characteristic of the research, as we only deal with the people of the Slovak public. The final limiting factor is the fact that the research was conducted right before the negative effects of the COVID-19 pandemic became evident, as the changes in public perception of nature during this time in Slovakia are already proven (Pichlerová et al., 2021). Following the COVID-19 pandemic, the military conflict (and subsequent economic and energy crises) taking place at the Slovak eastern border is bound to shift public perception of various issues, and this does not exclude environmental problems. With this in mind, we see our paper as a keystone for further research on these issues, as it offers the possibility of repeating the survey and discovering the effects of the COVID-19 pandemic and the war in Ukraine on the environmental attitudes of the Slovak public.

This approach can also be used as a supporting tool in research given citizens’ preferences in forest ecosystem services, as usually most studies focused on this issue until now have aimed at determining the value that is assessed by citizens to the individual forest ecosystem services. Using this approach, we can investigate deeper connections between personal preferences (in different areas) and the attitude toward the environment.

## 5 Conclusion

Following the objectives of the paper, we focused our attention on identifying the environmental attitudes of the Slovak public using the NEP approach. Based on our analyses, we managed to identify three slightly altered environmental attitudes among the Slovak public. We find this deviation from the NEP methodology to be acceptable, as environmental attitude is a very delicate set of values and views encompassing many different, yet significantly interrelated facets.

The Slovak public proved to be pro-environmentally oriented, as the majority of our respondents profess an ecocentric environmental attitude. Therefore, we can argue that the Slovak public mostly perceives humans to be an equally important part of nature, and care strongly about the quality of the environment, which suggests their inclination towards pro-environmental and sustainability-promoting political strategies, concepts, and so forth.

The NEP has proven to be a significant predictive method in the assessment of public environmental orientation, yet we recommend combining this method with an analysis of socio-demographic factors, as this would ensure a more comprehensive overview of public attitudes and perceptions of various issues.

## Data availability statement

The participants of this study did not give written consent for their data to be shared publicly, so due to the sensitive nature of the research supporting data is not publicly available. The data that support the findings of this study are available on request from the corresponding author, [JV].

## Ethics statement

Ethical review and approval was not required for this study in accordance with the local legislation and institutional requirements. The study was conducted in accordance with the local legislation and institutional requirements. Written informed consent for participation was not required from the participants or the participants’ legal guardians/next of kin in accordance with the national legislation and institutional requirements. At the beginning of the questionnaire, a statement regarding the processing and protection of respondents’ data was provided. Each participant who completed the questionnaire was informed and consented to the processing of their data by completing the questionnaire itself.

## Author contributions

LM: Conceptualization, Data curation, Investigation, Supervision, Writing – original draft, Writing – review & editing. MŠ: Conceptualization, Resources, Writing – original draft, Writing – review & editing. JV: Conceptualization, Formal analysis, Methodology, Visualization, Writing – review & editing. IH: Conceptualization, Resources, Writing – review & editing. BG: Conceptualization, Data curation, Investigation, Writing – review & editing. RŠ: Conceptualization, Resources, Writing – review & editing. ZS: Conceptualization, Resources, Writing – review & editing. JŠ: Conceptualization, Resources, Supervision, Writing – review & editing.
